# Simultaneous Removal of *Microcystis aeruginosa* and 2,4,6-Trichlorophenol by UV/Persulfate Process

**DOI:** 10.3389/fchem.2020.591641

**Published:** 2020-11-04

**Authors:** Jingwen Wang, Ying Wan, Siyang Yue, Jiaqi Ding, Pengchao Xie, Zongping Wang

**Affiliations:** ^1^School of Environmental Science and Engineering, Huazhong University of Science and Technology, Wuhan, China; ^2^School of Architecture & Urban Planning, Huazhong University of Science and Technology (HUST), Wuhan, China; ^3^Center for the Environmental Implications of Nanotechnology (CEINT), Durham, NC, United States; ^4^Hubei Provincial Engineering Research Center for Water Quality Safety and Pollution Control, Huazhong University of Science and Technology, Wuhan, China; ^5^Key Laboratory of Water & Wastewater Treatment (MOHURD), Huazhong University of Science and Technology, Wuhan, China

**Keywords:** *Microcystis aeruginosa*, 2,4,6-trichlorophenol, ultraviolet/persulfate, advanced oxidation process, cell integrity

## Abstract

UV/persulfate (UV/PS) could effectively degrade algal cells and micro-organic pollutants. This process was firstly applied to remove *Microcystis aeruginosa* (*M. aeruginosa*) and 2,4,6-trichlorophenol (TCP) simultaneously in bench scale. Algal cells can be efficiently removed after 120 min reaction accompanied with far quicker removal of the coexisted TCP, which could be totally removed within 5 min in the UV/PS process. Both ${\text{SO}}_{4}^{•-}$ and HO^•^ were responsible for algal cells and TCP degradation, while ${\text{SO}}_{4}^{•-}$ and HO^•^ separately dominated TCP degradation and algal cells removal. Apart from the role of radicals (${\text{SO}}_{4}^{•-}$ and HO^•^) for algal cells and TCP degradation, UV also played a role to some extent. Increased PS dose (0–4.5 mM) or UV intensity (2.71–7.82 mW/cm^2^) could enhance the performance of the UV/PS process in both TCP and algae removal. Although some intracellular organic matters can be released to the outside of algal cells due to the cell lysis, they can be further degraded by UV/PS process, which was inhibited by the presence of TCP. This study suggested the good potential of the UV/PS process in the simultaneous removal of algal cells and micro-organic pollutants.

**Graphical Abstract:**
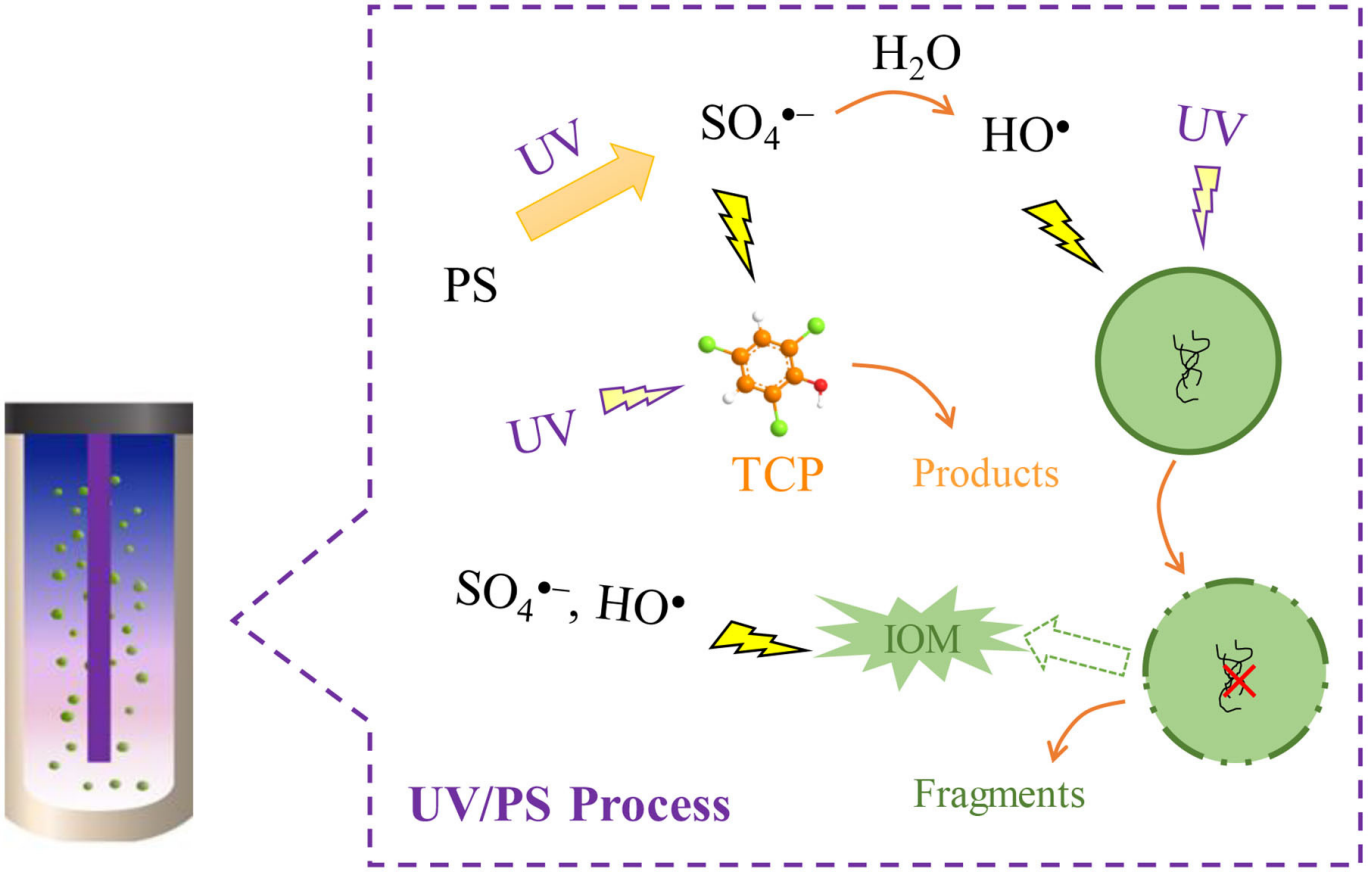
Principles of UV/PS process for the removal of *M. aeruginosa* and TCP.

## Introduction

Cyanobacterial blooms in lakes, urban ponds, and reservoirs have been frequently reported in recent years (Wang and Wei, 2008; de la Cruz et al., 2017; Keith et al., 2018). Bloom-forming cyanobacterial taxa are harmful to the environment and human health via consuming oxygen in water and releasing algal organic matter (AOM), such as toxins and taste & odor substances (Wang et al., 2016; Chen et al., 2017a). Additionally, the proliferation of algal cells can interrupt the supply of drinking water systems such as clogging filters and increasing the usages of treatment agent (Paerl and Otten, 2013; Xie et al., 2016). Due to the electrostatic repulsion, surface hydrophilicity, and steric effects of algal cells (Chow et al., 1999; Teixeira and Rosa, 2006; Shen et al., 2011), the conventional drinking water systems posed limited efficiency on the cells removal, causing serious deterioration in water quality (Rajasekhar et al., 2012). It is known that the discharge of municipal, industrial and agricultural wastewater which contains large amounts of micro-organic pollutants (MPs) can result in the occurrence of eutrophication and algal blooms (Liu et al., 2012). Therefore, natural water may be contaminated by algae and MPs at the same time, and it is meaningful to develop treatment methods to simultaneously remove algal cells and MPs.

Advanced oxidation processes (AOPs) could effectively degrade numerous MPs due to the production of high amounts of reactive radicals such as hydroxyl and sulfate radicals (HO^•^ and ${\text{SO}}_{4}^{•-}$) (Olmez-Hanci et al., 2015; Matzek and Carter, 2016). Due to the efficient destruction of algal cells by the formed reactive radicals, AOPs also perform well in removing algal cells (Wang et al., 2016; Wacławek et al., 2017; Chen et al., 2020). Consequently, the application of AOPs would simutaneously achieve good removal of algal cells and MPs in therory.

Among the reported AOPs, activation of persulfate (PS) by ultraviolet (UV) radiation can high-efficiently generate ${\text{SO}}_{4}^{•-}$ with a quantum yield of 1.4 mol E s^−1^ at 254 nm (Equation 1), accompanied with generating HO^•^ via Equation (2) (Mark et al., 1990; Xie et al., 2015). Both HO^•^ and ${\text{SO}}_{4}^{•-}$ are reactive radicals in the UV/PS process and account for the degradation of numerous MPs (Olmez-Hanci et al., 2015; Xie et al., 2015; Matzek and Carter, 2016). Furthermore, our previous studies have revealed that ultraviolet/persulfate (UV/PS) process is one of the most effective AOPs in the removal of algal cells and AOM (Wang et al., 2016; Chen et al., 2017a). So, the UV/PS process is expected to be a good choice in the simultaneous treatment of algal cells and MPs. Additionally, the released intracellular organic matter (IOM) during the treatment also shows high reactivity with reactive radicals with the second reaction rate constant between IOM and HO^•^ (*k*_IOM_) reaching (4.02~7.95) × 10^8^ Mc^−1^ s^−1^ (Lee et al., 2018), which would also scavenge the formed reactive radicals in the UV/PS process. Therefore, from a practical point of view, comprehensively investigating the treatability of co-existed algal cells and MPs by UV/PS would be of interest.

(1) $${\text{S}}_{2}{\text{O}}_{8}^{2-}\overset{\text{UV}}{\to }{2\text{SO}}_{4}^{•-}$$

(2) $${\text{SO}}_{4}^{•-}+{\text{H}}_{2}\text{O}\to {\text{HSO}}_{4}^{-}+{\text{HO}}^{•}$$

In this study, the UV/PS was firstly applied in removing algal cells and MPs simultaneously. The objective of this study is to evaluate the UV/PS performance on the removal of algae and MPs simultaneously by choosing the widely detective *Microcystis aeruginosa* (*M. aeruginosa*) and 2,4,6-trichlorophenol (TCP) as examples of typical algal cells and MP, respectively. The removal efficiencies of algal cells and TCP under different conditions were studied, and the roles of reactive radicals (i.e., HO^•^ and ${\text{SO}}_{4}^{•-}$) were evaluated. The variation of cells integrity and typical AOM characteristics were investigated. The interaction between algae and TCP, and some influencing factors were also assessed.

## Materials and Methods

### Materials

TCP (98%) was purchased from Aladdin (Shanghai, China), and its stock solution of 12 mM was prepared by pure water (Micropure UV, Thermo Fisher Scientific, USA) containing 0.01 mM NaOH. *M. aeruginosa* (No. FACHB-909) was obtained from the Institute of Hydrobiology, Chinese Academy of Sciences and cultured in BG-11 media according to our previous research (Wang et al., 2016). Cells were harvested by centrifugation at 4,500 rpm for 10 min and re-suspended with 15 mM NaClO_4_ solution twice to remove residual BG-11 media. Then the algae solution was diluted to 1 × 10^6^ cells/mL by adding 15 mM NaClO_4_ solution (Liu et al., 2018). All the other chemical reagents of analytical grade at least were obtained from Sinopharm Chemical Reagent Co., Ltd. (Shanghai, China).

### Experimental Procedures

For the UV/PS system, a 600 mL cylindrical glass vessel equipped with a low-pressure mercury UV lamp (254 nm, GPH135T5 L/4, Heraeus Noblelight) was used following our previous research (Supplementary Figure 1; Wang et al., 2016). Three different UV intensities (2.71, 5.37, and 7.82 mW/cm^2^) were obtained through wrapping the UV lamp with copper wire cloth and measured by the photolysis of H_2_O_2_ (Li et al., 2012). The UV reactor was thermo-stated (DC-0510, Hannuo, China) at 25 ± 1°C and switched on at least 15 min before the reaction. Na_2_S_2_O_8_ powder was added to the prepared solution containing both algae and TCP, then the solution was quickly transferred into the UV reactor after adjusting the pH by 0.1 M HClO_4_ or NaOH. After the reaction, samples were collected at predetermined time intervals and quenched using excess sodium thiosulfate for further measurement.

### Analytical Methods

The absorbance of *M. aeruginosa* at 680 nm (OD_680_) was read by a UV-vis spectrophotometer (U-3100PC, Mapada, Shanghai, China), which was linear with cell density with *R*^2^ of 0.996 in this study (Supplementary Figure 2; Wang et al., 2016). TCP was first acetylated with acetic anhydride in the presence of K_2_CO_3_ and then measured by a gas chromatography (GC-2014C, Shimadzu, Japan) equipped with a ZB-5 column (30 m × 0.25 mm, ID 0.25 μm) and an electron capture detector (ECD) (Rodríguez et al., 1996). Chromatographic parameters include 200°C injector temperature, 290°C ECD temperature, and 160°C oven temperature.

Other parts of the samples were filtered through 0.45 μm cellulose acetate membranes and characterized by fluorescence excitation-emission matrix (EEM) using an F-4600 fluorescence spectrophotometer (Hitachi, Japan). The PS concentration was measured by a rapid spectrophotometric method according to a previous research (Liang et al., 2008). A flow cytometer (FCM) (Guava easyCyte5, Amnis Merck Millipore, USA) at a fixed wavelength of 488 nm was employed to evaluate the cell breakage during reaction. SYTOX Green nucleic acid stain (Life Technologies, US) was used to determine the percentages of viable and non-viable cells (Xie et al., 2013; Sun et al., 2018). In detail, 0.5 mL sample was stained with 50 nM SYTOX Green nucleic acid stain and incubated for 10 min at room temperature. The settings used for FCM were: flow rate mode = very low, FSC = 10^0^ – 10^5^, SSC = 10^0^ – 10^5^, Number of events = 5,000. The Green fluorescence (530 nm) and the red fluorescence (630 nm) were recorded in channel Green-B and Red-B, respectively.

## Results and Discussion

### Comparison of UV, PS, and UV/PS Processes on Algae and TCP Removal

**Figure 1:**
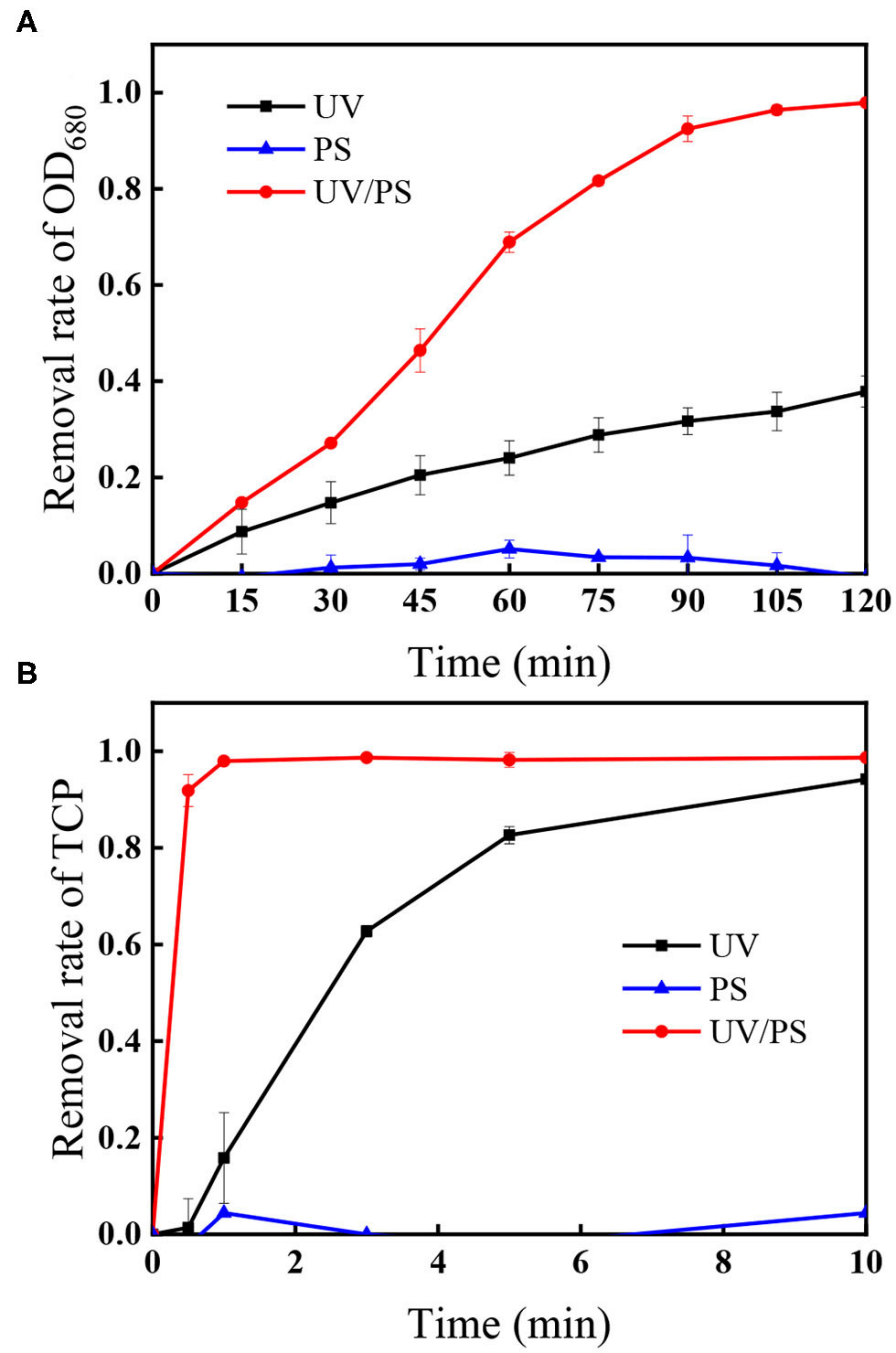
The removal of **(A)** OD_680_ and **(B)** TCP under different treatment processes. Conditions: initial algal cell density: (1.00 ± 0.05) × 10^6^ cells/mL, initial TCP concentration: 20 μM, initial pH: 7.6 ± 0.2, temperature: 25 ± 1°C, UV intensity: 7.82 mW/cm^2^, PS dose: 4.5 mM. The error bars represent the standard deviations from duplicate tests.

Figure 1 shows the removal of algae and TCP under UV irradiation, PS oxidation, and UV/PS oxidation. Negligible removal of algae or TCP was observed in the presence of 4.5 mM PS, indicating that PS could not effectively remove algae or TCP through direct oxidation. When the solution was treated by UV irradiation, 37.8% of algae was removed after 120 min, and the coexisted TCP was rapidly degraded by 94.1% within 10 min. Among all the selected processes, UV/PS showed the best performance on algae and TCP removal as 97.9% of algal cells and 98.7% of the coexisted TCP were removed after reaction. The coexisted TCP was removed much faster (98.7% removal within 10 min) than algal cells in the UV/PS process, indicating that TCP was more vulnerable when it coexisted in algae solution. Although both UV and UV/PS processes can efficiently degrade TCP, the TCP degradation rate in the UV/PS process was far quicker than that in the UV alone. The aforementioned results suggest that the formed reactive radicals in the UV/PS process were believed to account for the removal of algal cells and TCP, while direct UV radiation would also play an important role in TCP degradation.

### Roles of Sulfate and Hydroxyl Radicals in the UV/PS Process

**Figure 2:**
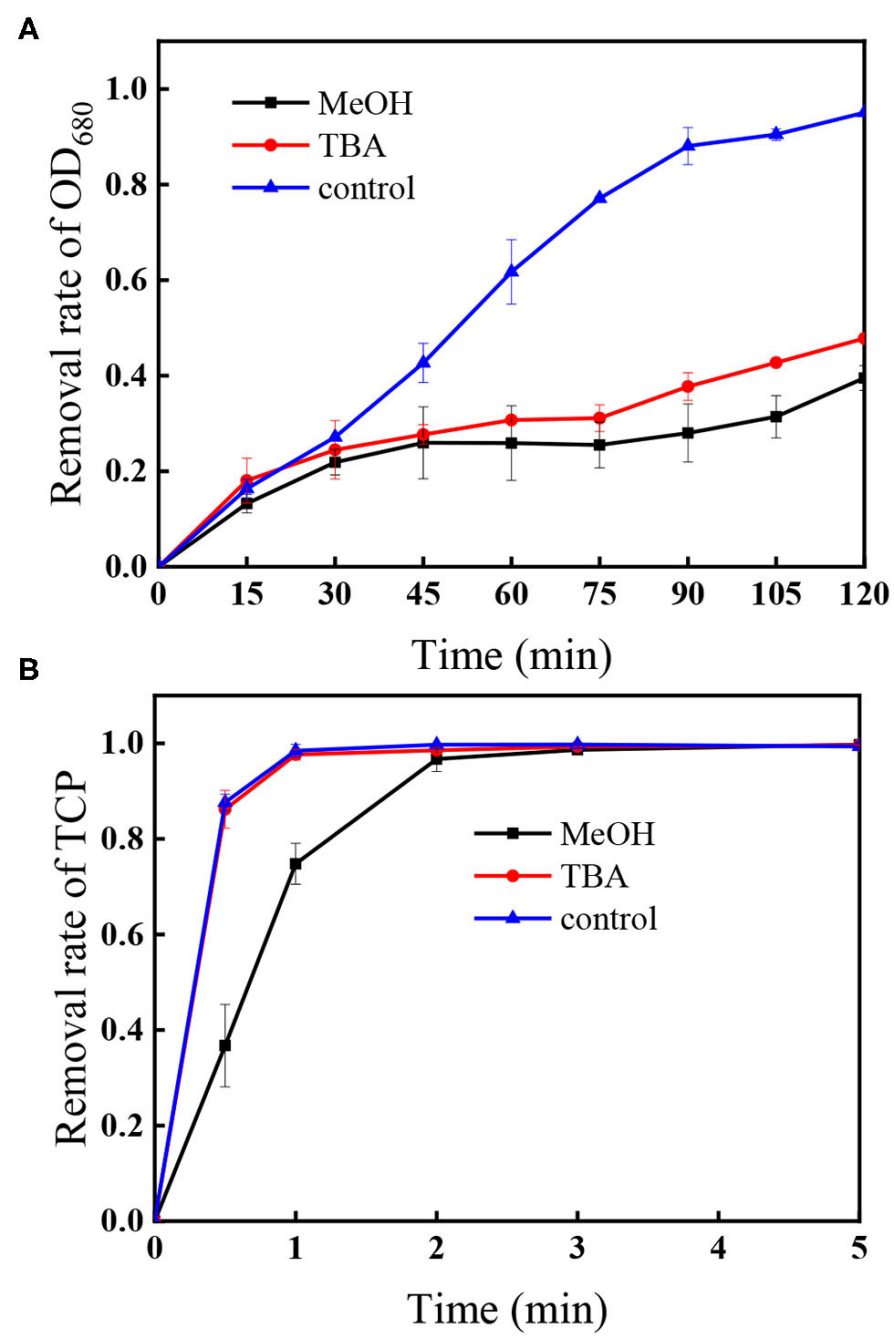
Impacts of TBA and MeOH on the removal of **(A)** OD_680_ and **(B)** TCP. Conditions: initial algal cell density: (1.00 ± 0.05) × 10^6^ cells/mL, initial TCP concentration: 20 μM, initial pH: 7.6 ± 0.2, temperature: 25 ± 1°C, UV intensity: 7.82 mW/cm^2^, PS dose: 1.5 mM, [MeOH] = [TBA] = 22.5 mM. The error bars represent the standard deviations from duplicate tests.

In the UV/PS process, the photolysis of PS generates ${\text{SO}}_{4}^{•-}$ and HO^•^ via Equation (1) and (2) (Mark et al., 1990; Xie et al., 2015). Thus, HO^•^ and ${\text{SO}}_{4}^{•-}$ are usually the main reactive radicals in the UV/PS process, which would be responsible for the degradation of algal cells and TCP. To confirm the roles of HO^•^ and ${\text{SO}}_{4}^{•-}$, *tert*-butyl alcohol (TBA) and methanol (MeOH) were applied as specific scavengers. MeOH can scavenge both HO^•^ and ${\text{SO}}_{4}^{•-}$ with the second reaction rate constant of 1.1 × 10^7^ M^−1^s^−1^ for ${\text{SO}}_{4}^{•-}$ and 9.7 × 10^8^ M^−1^s^−1^ for HO^•^, respectively (Buxton et al., 1988; Neta et al., 1988). As for TBA, the second reaction rate constant for HO^•^ (6.0 × 10^8^ M^−1^s^−1^) is 1,500-fold greater than that for ${\text{SO}}_{4}^{•-}$ (4.0 × 10^5^ M^−1^s^−1^), suggesting that it is only a good scavenger of HO^•^ (Buxton et al., 1988; Neta et al., 1988). Figure 2A displays the changes of algae within 120 min in the UV/PS process, and the removal efficiencies of algae in the presence of TBA or MeOH were 47.8 and 39.5%, respectively. Comparing with the removal rate without the presence of scavengers (control in Figure 2A), it could be concluded that the algae removal is mainly attributed to HO^•^ and UV irradiation (Figure 1A). Although ${\text{SO}}_{4}^{•-}$ also took part in the removal of algal cells, it was not the dominant reactive species in algae oxidation, which could be explained by the fact that ${\text{SO}}_{4}^{•-}$ shows lower reactivity toward microbes compared to HO^•^ (Sun et al., 2016). Furthermore, the zeta potential of *M. aeruginosa* ranges from −25 to −30 mV at the selected pH (7.0), which would decrease the contact possibility between the algal cells and negatively charged ${\text{SO}}_{4}^{•-}$ (Henderson et al., 2008; Chen et al., 2017a; Liu et al., 2017).

As shown in Figure 2B, the degradation of TCP in the presence of TBA in the UV/PS process followed the same trend as that without TBA (control in Figure 2B), while the addition of MeOH can efficiently slow down the degradation rate of TCP in the process. As TBA can scavenge HO^•^ but has negligible effect on ${\text{SO}}_{4}^{•-}$ (Chen et al., 2017b), this result suggests that the coexisted TCP could be degraded by ${\text{SO}}_{4}^{•-}$ and direct UV irradiation.

### Interaction Between Algae and TCP

As discussed above, the main reactive radicals oxidizing algal cells and TCP were HO^•^ and ${\text{SO}}_{4}^{•-}$, respectively, which left the question of the possible interaction of these radicals with TCP and algal cells. Supplementary Figure 3A shows the removal of TCP under different initial TCP concentrations in the UV/PS process. As expected, an increase in the initial TCP concentration causes a decrease in TCP degradation. When TCP concentration increased from 5 to 20 μM, the degradation efficiencies decreased from 97.5 to 64.2% in 0.5 min. Furthermore, the presence of TCP could decrease algae removal somewhat, with the removal of algae decreased by about 13.3% after 60 min in the presence of 20 μM TCP compared to the sample in the absence of TCP (Supplementary Figure 3B). The scavenging effect of TCP on ${\text{SO}}_{4}^{•-}$ and further inhibiting the generation of HO^•^ via Equation (2) were expected to be responsible for the inhibition of algae removal (Xie et al., 2015).

Supplementary Figure 4A shows similar final algal cell density after reaction for 120 min in the presence of 0.75 mM PS even the initial algal cells were doubled with the initial OD_680_ increasing from 0.02 to 0.04, suggesting that higher initial algae concentration could accelerate the removal of algal cells in the system. The possible reason was that higher concentration of algal cells would compete more reactive radicals. However, the different initial algal density showed little effect on TCP degradation, which could be explained by the fact that the initial generated ${\text{SO}}_{4}^{•-}$ only had small reactivity toward algal cells (Figure 2).

### EEM Spectra of Released IOM and Cell Integrity

The fluorescence EEM spectra of released IOM under different treatment processes including UV alone, PS and UV/PS oxidation are shown in Supplementary Figure 5. IOM was not be released into solutions in both untreated and PS oxidation processes as there were no obvious fluorescence signal observed in Supplementary Figures 5A,B. After UV irradiation for 30 min, four EEM peaks including peak I (Ex/Em ~ 350/432 nm, represents humic acid-like organics), peak II (Ex/Em ~ 270/442 nm, represents fulvic acid-like materials), peak III (Ex/Em ~ 230/330 nm, represents simple aromatic proteins), and peak IV (Ex/Em ~ 280/330 nm, represents soluble microbial byproduct-like materials) suggested that IOM were released from cells by UV treatment (Supplementary Figure 5C; Chen et al., 2003). All of these peaks disappeared after UV/PS oxidation (Supplementary Figure 5D), suggesting that the released IOM would be further oxidized by the generated radicals. Additionally, the presence of 20 μM TCP did not affect the fluorescence of IOM in the UV/PS process after 30 min (Supplementary Figure 5E), which might attribute to the fast removal of TCP with a relatively high dose of PS (1.5 mM).

**Figure 3:**
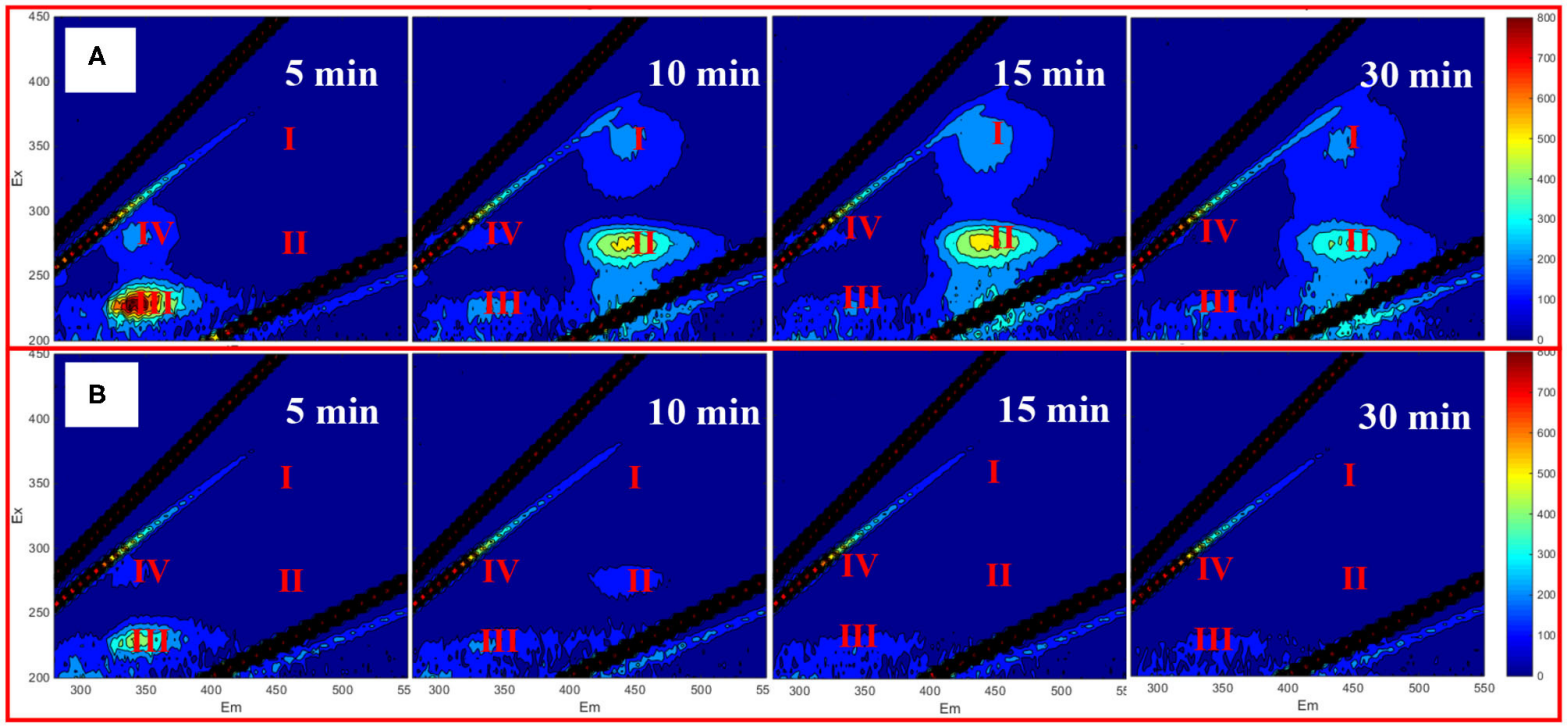
EEM of extracellular AOM with 20 μM TCP **(A)** and without TCP **(B)**. Conditions: initial algal cell density: (1.00 ± 0.05) × 10^6^ cells/mL, initial pH: 7.6 ± 0.2, temperature: 25 ± 1°C, UV intensity: 7.82 mW/cm^2^, PS dose: 0.05 mM. The error bars represent the standard deviations from duplicate tests.

To further understand the performance of EEMs spectra of IOM in the presence of TCP, PS was decreased to 0.05 mM. Figure 3 depicts the EEMs spectra of the released IOM as a function of reaction time in the UV/PS process with or without TCP, and no obvious peaks were observed in the absence of TCP (Figure 3A). It has reported that the IOM from *M. aeruginosa* could be fast oxidized by reactive radicals (Lee et al., 2018), which can explain the no obvious peaks during the treatment by UV/PS in the absence of TCP. In contrast, when TCP coexisted in the algae solution, peaks IV and III were first observed to be enhanced after 5 min reaction and then began to disappear when the oxidation time was over 10 min, suggesting that the presence of TCP could retard the oxidation of the released IOM in the UV/PS process (Figure 3A). Additionally, although no peaks in areas I and II were observed after oxidation for 5 min, significant peaks appeared in the two areas after treatment for 10 min, and their intensities followed a decreasing trend with further prolonging treatment time. The aforementioned results suggested that the released IOM contained numerous protein-like fractions which can be transferred to humic and fulvic acid-like compounds by UV/PS treatment. Then the formed humic and fulvic acid-like compounds can be further oxidized or even mineralized by the reactive radicals in the UV/PS process. These results are partly in line with some previous studies majoring in the treatment of algae-containing water by UV-based advanced oxidation processes (Wang et al., 2016; Sun et al., 2018; Chen et al., 2020).

**Figure 4:**
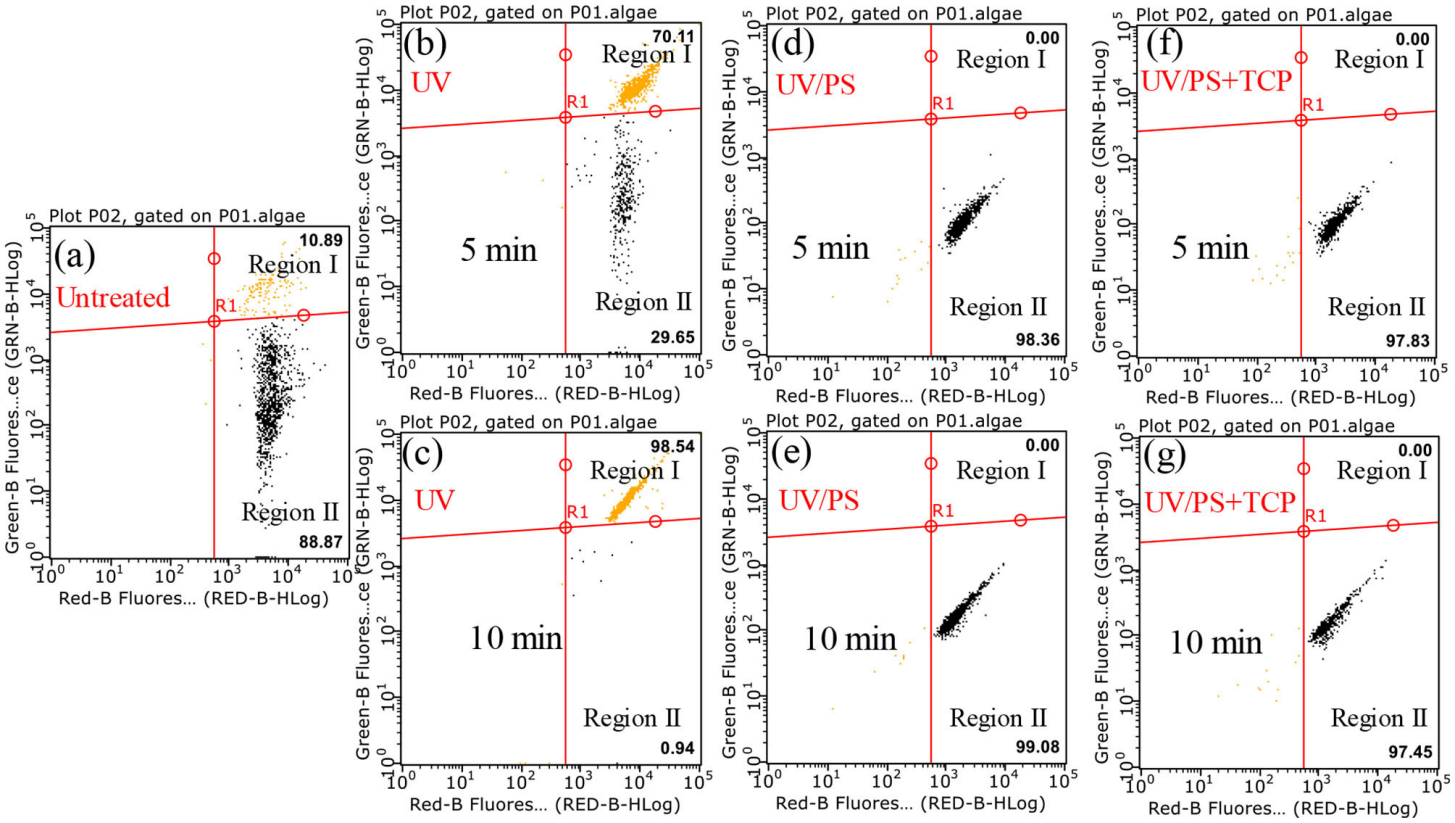
Flow cytometry results after treatment by different processes. Conditions: initial algal cell density: (1.00 ± 0.05) × 10^6^ cells/mL, initial TCP concentration: 0 μM for **(a–e)** and 20 μM for **(f,g)**, initial pH: 7.6 ± 0.2, temperature: 25 ± 1°C, UV intensity: 7.82 mW/cm^2^, PS dose: 1.5 mM. The error bars represent the standard deviations from duplicate tests.

Figure 4 presents the flow cytometry results under UV and UV/PS treatment, and SYTOX green nucleic acid stain was used to distinguish the viable and non-viable cells. The RED-B and Green-B represent chlorophyll auto-fluorescence and cell permeability, respectively (Daly et al., 2007; Xie et al., 2013). Region I (SYTOX Green positive) and Region II (SYTOX green negative) were associated with the percentages of the non-viable and viable cells. As shown in Figure 4a, most of the algae are viable cells in the untreated sample as 88.87% of cells showed SYTOX green negative in Region II. Compared with the untreated sample, the proportion of viable cells decreased from 88.87 to 29.65% after UV irradiation for 5 min and further decreased to 0.94% within 10 min, indicating that UV irradiation played significant roles in cell destruction. However, the proportion of cells in Region II increased again (98.36% in 5 min and 99.08% in 10 min) in the UV/PS process (Figures 4d,e), which was similar to a previous research (Wang et al., 2016). This phenomenon could be explained by the fact that the nucleic acid in cells was further oxidized by the generated ${\text{SO}}_{4}^{•-}$ and HO^•^ as no signals from nucleic acid stained by SYTOX were found in Region I (Figures 4d,e; Daly et al., 2007). Additionally, there are no signals in Region I when TCP was added in the solution in the UV/PS process (Figures 4f,g), which suggested that the presence of TCP cannot retard the nucleic acid oxidation at the given reaction conditions.

### Effects of PS Dose and UV Intensity

**Figure 5:**
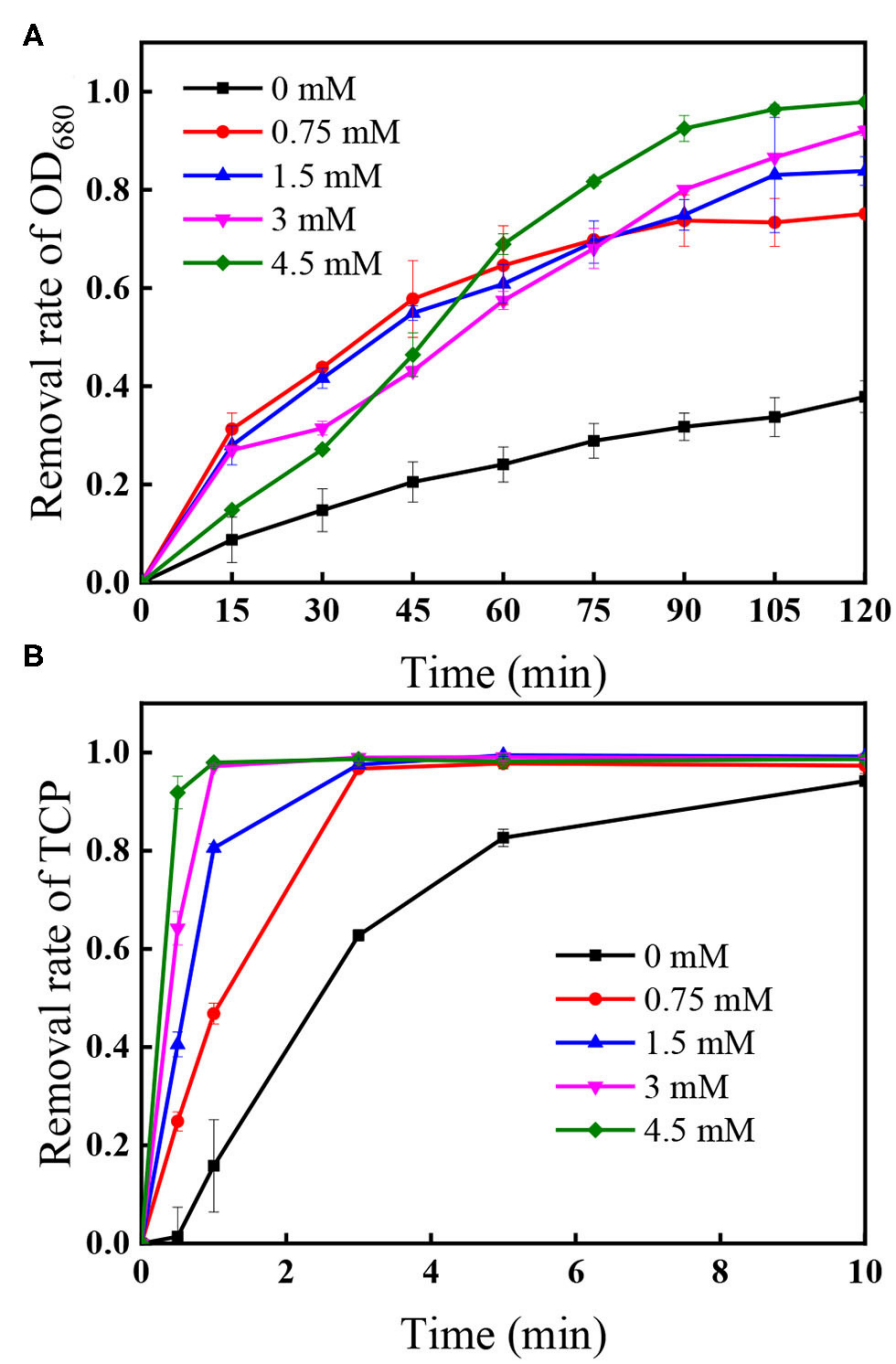
Effect of PS dose on the removal of **(A)** OD_680_ and **(B)** TCP. Conditions: initial algal cell density: (1.00 ± 0.05) × 10^6^ cells/mL, initial TCP concentration: 20 μM, initial pH: 7.6 ± 0.2, temperature: 25 ± 1°C, UV intensity: 7.82 mW/cm^2^. The error bars represent the standard deviations from duplicate tests.

Figure 5 presents the effect of PS concentration (0.75–4.5 mM) on algal cells and TCP degradation in the UV/PS process, and the removal efficiency of algal cells and TCP increased from 75.5 to 97.9% after 120 min (Figure 5A) and from 46.8 to 97.9% within 1 min (Figure 5B), respectively. In the selected PS range, the coexisted TCP was quickly degraded within 5 min (removal rate >97%). Initial PS concentration is critical in UV/PS process as PS is the source of ${\text{SO}}_{4}^{•-}$ (Equation 1), as a result to promoting the removal of algae and the degradation of micro-organic pollutants with elevating PS dosage (Xie et al., 2015; Chen et al., 2017b). As nearly all the cells showed non-viable after 10 min UV irradiation and nucleic acid was totally destroyed within 5 min in the UV/PS process (Figure 4), the algal cells must underwent an irreversible rupture by UV/PS oxidation, and deposed to fragments after that.

Supplementary Figure 6 shows the PS decomposition under different initial PS concentration followed the first-order kinetics in the selected PS range, indicating that the presence of algal cells and TCP would not affect the first-order photolysis of PS significantly. Additionally, the residual PS after 120 min UV irradiation was <2% in each selected initial PS dose, suggesting that few PS would left after applying UV/PS to simultaneously treat algae and TCP.

**Figure 6:**
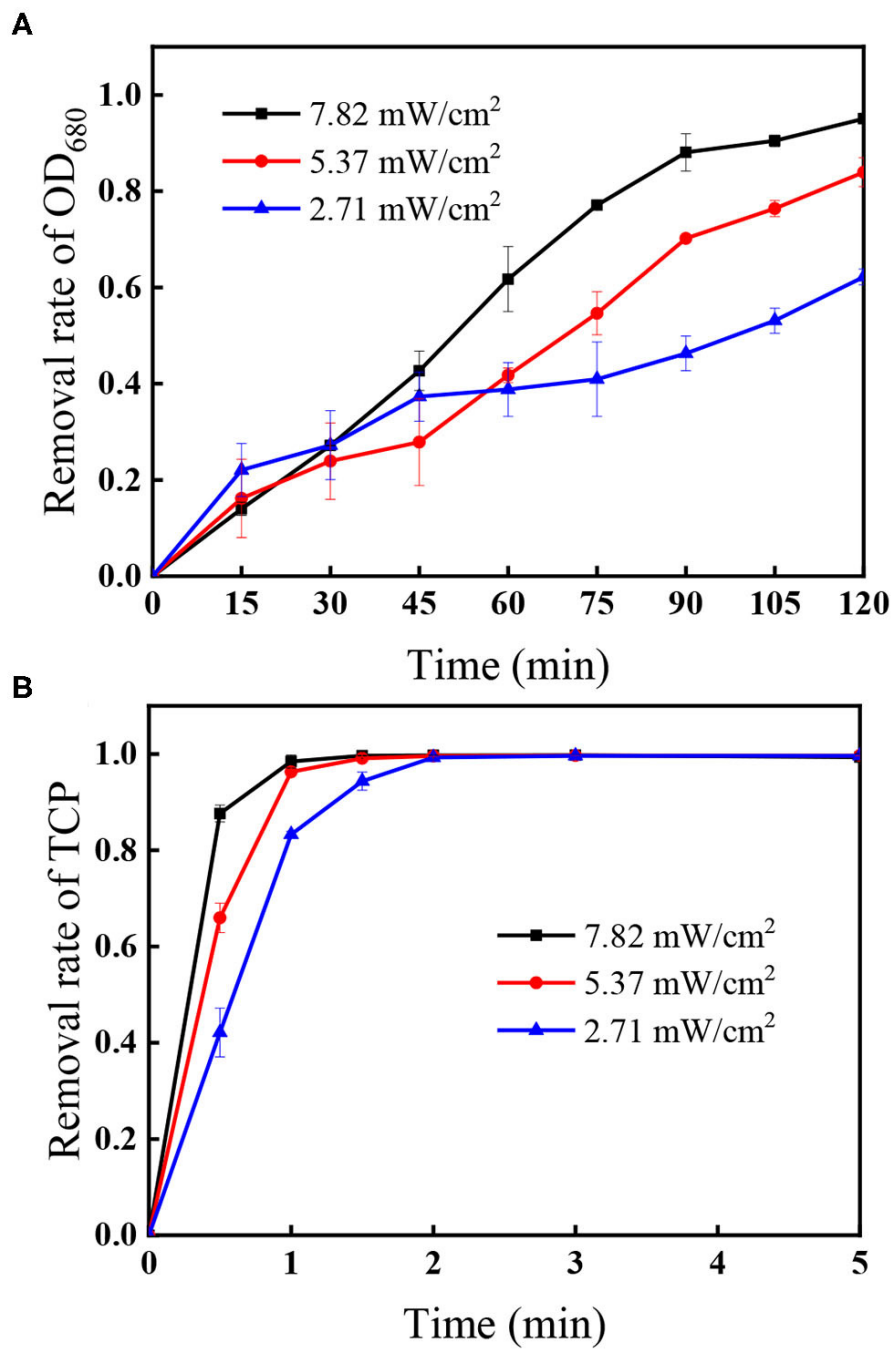
Effect of UV intensity on the removal of **(A)** OD_680_ and **(B)** TCP. Conditions: initial algal cell density: (1.00 ± 0.05) × 10^6^ cells/mL, initial TCP concentration: 20 μM, initial pH: 7.6 ± 0.2, temperature: 25 ± 1°C, PS dose: 1.5 mM. The error bars represent the standard deviations from duplicate tests.

Figure 6 shows that the removal of algal cells increased from 62.2 to 95.0% when UV intensity increased from 2.71 to 7.82 mW/cm^2^ within 120 min, accompanied with rapid decomposition efficiencies of the coexisted TCP increasing from 83.3 to 98.4% within 1 min. As the radical quantum yields of PS under UV_254_ activation is 1.4 (Crittenden et al., 1999), more reactive radicals including ${\text{SO}}_{4}^{•-}$ and HO^•^ would be generated under higher UV intensity (Xie et al., 2015). Additionally, Figure 1 shows that direct photolysis also played important role in the removal of algae and the TCP degradation, meaning that higher photon intensity can also improve the direct photolysis efficiency.

## Conclusion

In this study, UV/PS process was firstly applied to simultaneously treat algal cells (*M. aeruginosa*) and TCP. ${\text{SO}}_{4}^{•-}$ and direct UV photolysis dominated TCP degradation, while HO^•^ and UV direct photolysis were evidenced to play the primary roles in algae removal. Increasing PS dose and UV intensity could enhance both TCP and algae removal. Higher initial TCP concentration (20 μM) can significantly slow down the removal of algal cells and TCP, but high final treatment efficiency was always achieved. In the selected algal densities (OD_680_ = 0.02, 0.04), initial algae densities played little role in the remained OD_680_ and TCP concentration after UV/PS treatment. Although IOM were released from the lysed algal cells in the UV/PS process, they could be further oxidized by the generated reactive radicals. Due to the competition of generated reactive radicals, the presence of TCP could inhibit IOM removal in the UV/PS process. However, the inhibitory effect could be neglected at high PS dose (>1.5 mM). After reaction for 120 min in the selected experimental conditions, the residual PS was <2%, ensuring the safety of UV/PS treatment strategy to degrade TCP and algae.

## Data Availability Statement

All datasets generated for this study are included in the article/Supplementary Material.

## Author Contributions

JW: conceptualization, data curation, writing-original draft preparation, formal analysis, and investigation. JD, SY, and YW: writing-reviewing. PX: conceptualization, co-supervision, writing-reviewing and editing, project administration, formal analysis, funding acquisition, and resources. ZW: co-supervision, writing-reviewing, formal analysis, and resources. All authors approved it for publication.

## Conflict of Interest

The authors declare that the research was conducted in the absence of any commercial or financial relationships that could be construed as a potential conflict of interest.
