# Supplementary material for: Simultaneous Removal of Microcystis aeruginosa and 2,4,6-Trichlorophenol by UV/Persulfate Process
Source: Front Chem. 2020 Nov 4;8:591641. doi: 10.3389/fchem.2020.591641 (PMC7673307; doi:10.3389/fchem.2020.591641)
Supplement: Supplementary file 1 [file Data_Sheet_1.pdf]

Supporting Information

for

Simultaneous removal of *Microcystis aeruginosa* and 2, 4, 6-trichlorophenol by UV/persulfate process

Jingwen Wang [a], Ying Wan [a], Siyang Yue [b], Jiaqi Ding [a], Pengchao Xie [a,c,d*], Zongping Wang [a,d,e*]

[a] *School of Environmental Science and Engineering, Huazhong University of Science and Technology, Wuhan 430074, China*

[b] *School of Architecture & Urban Planning, Huazhong University of Science and Technology (HUST), Wuhan 430074, China*

[c] *Center for the Environmental Implications of Nanotechnology (CEINT), Durham 27708-0287, USA*

[d] *Hubei Provincial Engineering Research Center for Water Quality Safety and Pollution Control, Huazhong University of Science and Technology, Wuhan 430074, China*

[e] *Key Laboratory of Water & Wastewater Treatment (MOHURD), Huazhong University of Science and Technology, Wuhan 430074, China*

** Corresponding author: pengchao_xie@hust.edu.cn (Pengchao Xie), zongpingw@hust.edu.cn (Zongping Wang)*

Captions

Fig. S1. The diagram of UV/PS reactor 3

Fig. S2. The regression equation between OD_{680} (x) and cell density (y, $\times 10^6$ cells/mL). 4

Fig. S3. Effect of initial TCP concentration on the removal of (a) OD_{680} and (b) TCP. Conditions: initial algal cell density: $(1.00 \pm 0.05) \times 10^6$ cells/mL, initial pH: 7.6 ± 0.2, temperature: 25 ± 1 °C, PS dose: 1.5 mM. The error bars represent the standard deviations from duplicate tests 5

Fig. S4. Effect of initial algae densities on the removal of (a) OD_{680} and (b) TCP. Conditions: initial TCP concentration: 20 μM, initial pH: 7.6 ± 0.2, temperature: 25 ± 1 °C, PS dose: 0.75 mM. The error bars represent the standard deviations from duplicate tests 6

Fig. S5. The EEM of extracellular AOM after treatment by different processes . Conditions: initial algal cell density: $(1.00 \pm 0.05) \times 10^6$ cells/mL, initial TCP concentration: 0 μM for (a)–(d) and 20 μM for (e), initial pH: 7.6 ± 0.2, temperature: 25 ± 1 °C, UV intensity: 7.82 mW/cm^2, PS dose: 1.5 mM. 7

Fig. S6. PS decomposition under different PS dose. Insert: first-order kinetics of PS decomposition. Conditions: initial algal cell density: $(1.00 \pm 0.05) \times 10^6$ cells/mL, initial TCP concentration: 20 μM, initial pH: 7.6 ± 0.2, temperature: 25 ± 1 °C, UV intensity: 7.82 mW/cm^2. The error bars represent the standard deviations from duplicate tests 8

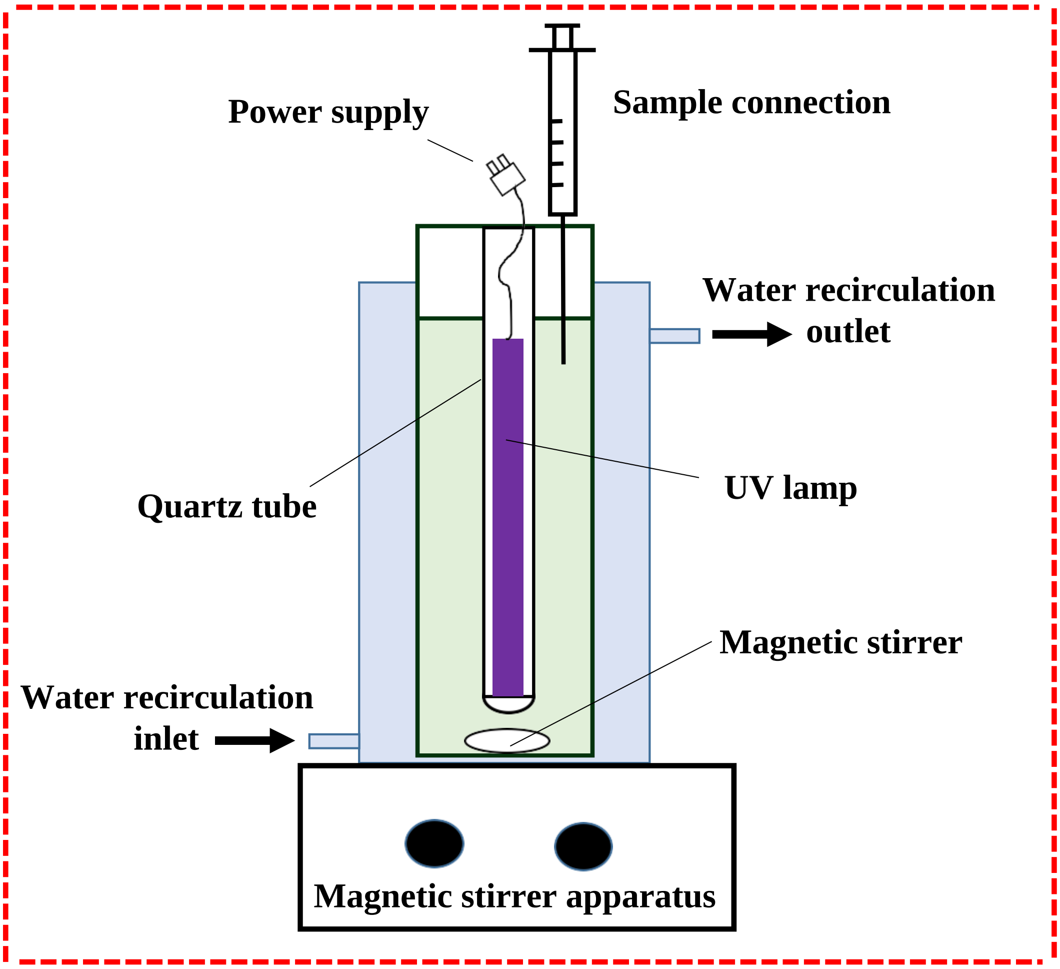

Fig. S1. The diagram of UV/PS reactor.

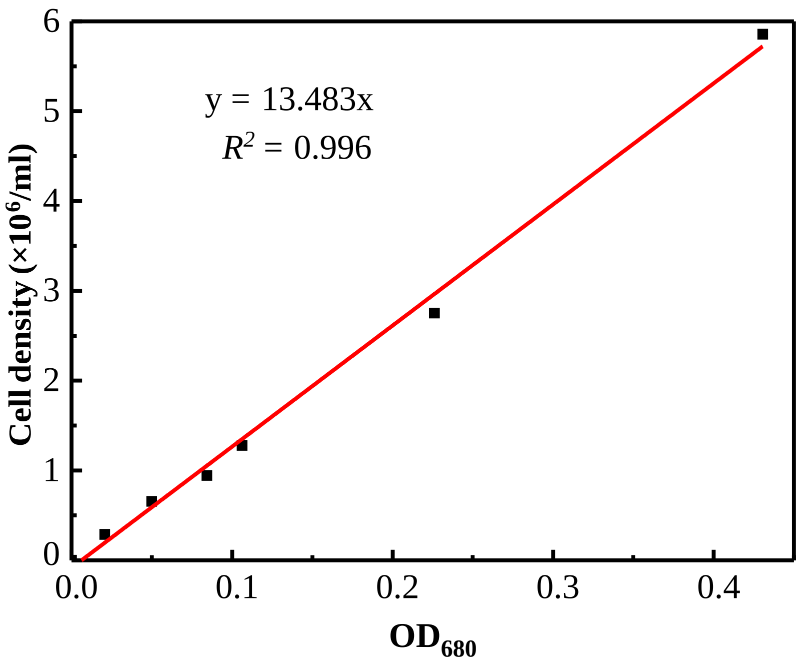

Fig. S2. The regression equation between OD_{680} (x) and cell density (y, $\times 10^6$ cells/mL).

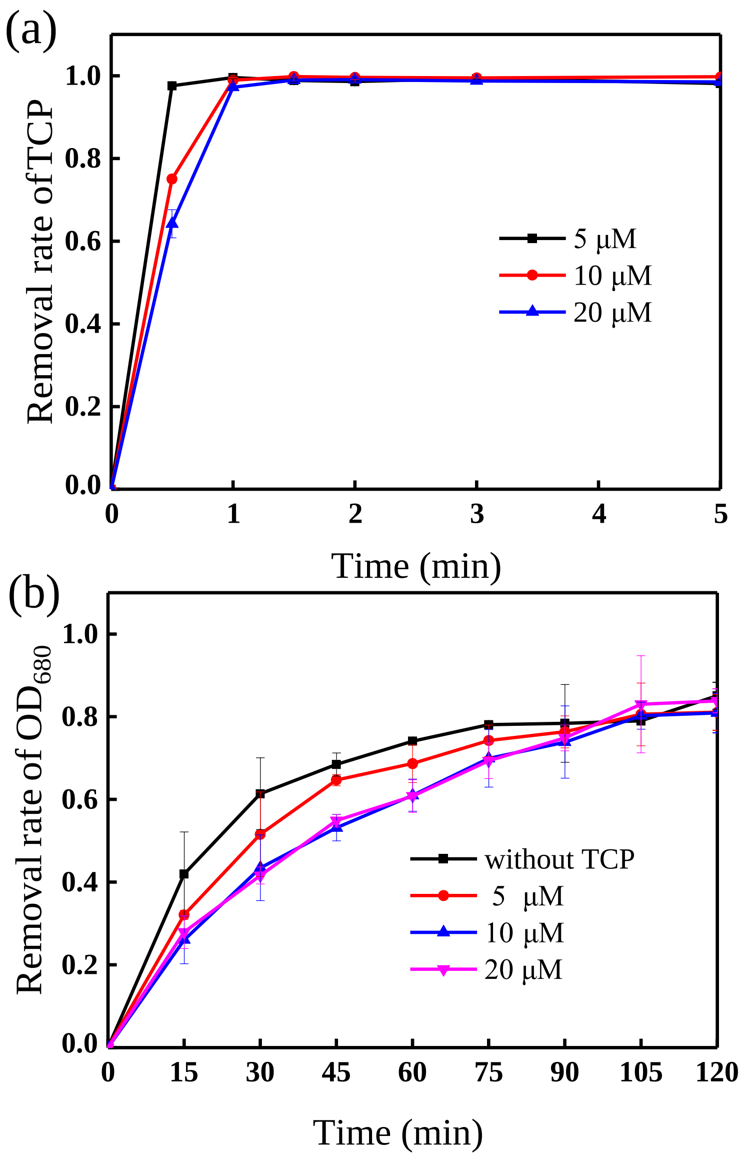

Fig. S3. Effect of initial TCP concentration on the removal of (a) OD_{680} and (b) TCP. Conditions: initial algal cell density: (1.00 ± 0.05) × 10^6 cells/mL, initial pH: 7.6 ± 0.2, temperature: 25 ± 1 °C, PS dose: 1.5 mM. The error bars represent the standard deviations from duplicate tests.

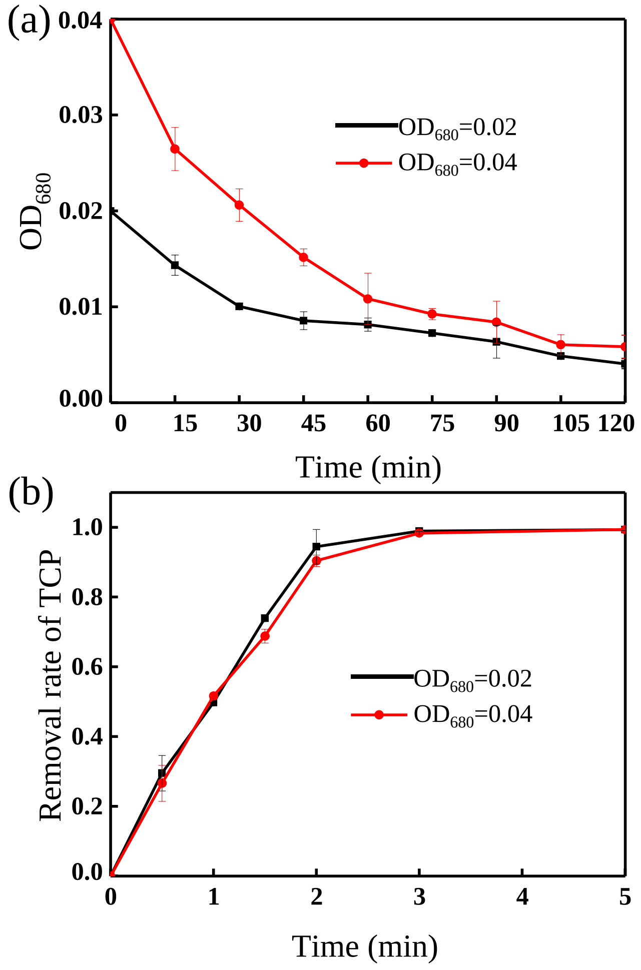

Fig. S4. Effect of initial algae densities on the removal of (a) OD_{680} and (b) TCP. Conditions: initial TCP concentration: 20 μM, initial pH: 7.6 ± 0.2, temperature: 25 ± 1 °C, PS dose: 0.75 mM. The error bars represent the standard deviations from duplicate tests.

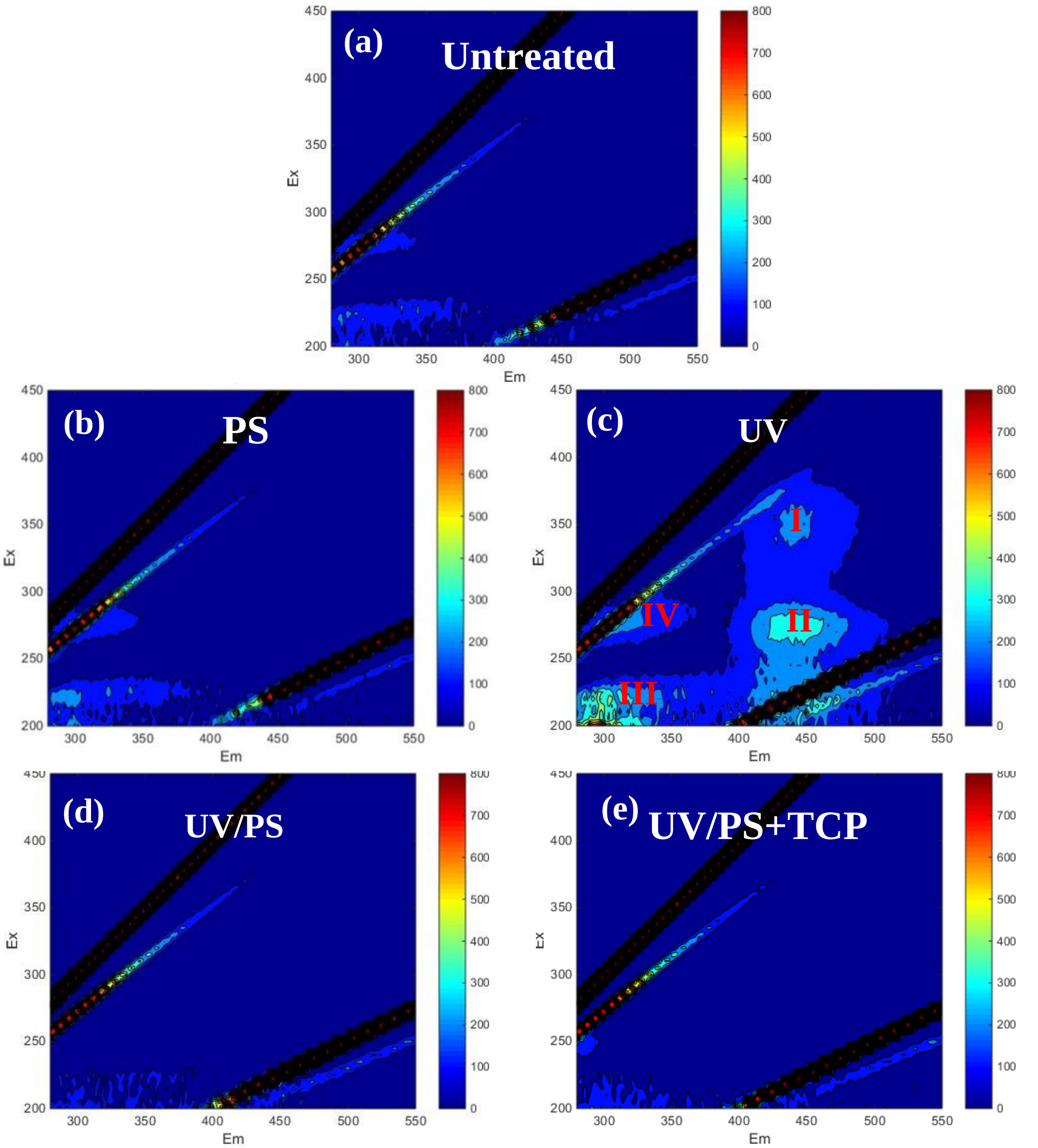

Fig. S5. The EEM of extracellular AOM after treatment by different processes . Conditions: initial algal cell density: (1.00 ± 0.05) × 10^6 cells/mL, initial TCP concentration: 0 μM for (a)–(d) and 20 μM for (e), initial pH: 7.6 ± 0.2, temperature: 25 ± 1 °C, UV intensity: 7.82 mW/cm^2, PS dose: 1.5 mM.

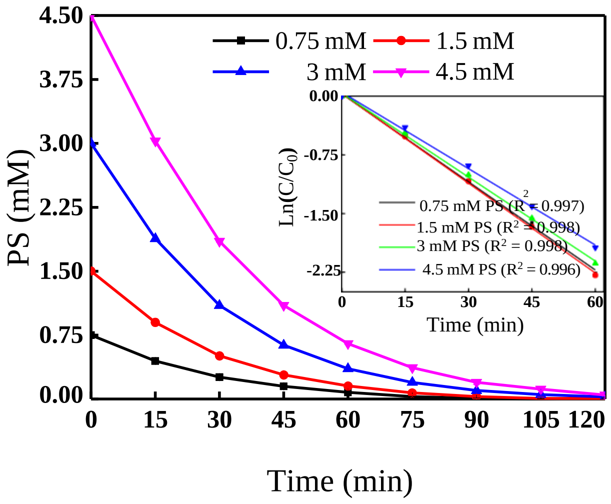

Fig. S6. PS decomposition under different PS dose. Insert: first-order kinetics of PS decomposition. Conditions: initial algal cell density: (1.00 ± 0.05) × 10^6 cells/mL, initial TCP concentration: 20 μM, initial pH: 7.6 ± 0.2, temperature: 25 ± 1 °C, UV intensity: 7.82 mW/cm^2. The error bars represent the standard deviations from duplicate tests.
